# Common Factor Structure of the Ten Subtest Wechsler Adult Intelligence Scale–Fourth Edition in a Clinical Sample and 15 Subtest Version in the Standardization Sample

**DOI:** 10.1093/arclin/acad035

**Published:** 2023-05-23

**Authors:** Navaneetham J Sudarshan, Stephen C Bowden

**Affiliations:** Melbourne School of Psychological Sciences, University of Melbourne, Parkville, Victoria, Australia; Melbourne School of Psychological Sciences, University of Melbourne, Parkville, Victoria, Australia; Department of Clinical Neurosciences, St. Vincent’s Hospital, Fitzroy, Victoria, Australia

**Keywords:** Factorial invariance, Generalizability, Validity, WAIS-IV

## Abstract

**Objective:**

The 10 core subtests of the Wechsler Adult Intelligence Scale-IV (WAIS-IV) suffice to produce the 4 index scores for clinical assessments. Factor analytic studies with the full complement of 15 subtests reveal a 5-factor structure that aligns with Cattell–Horn–Carroll taxonomy of cognitive abilities. The current study investigates the validity of 5-factor structure in a clinical setting with reduced number of 10 subtests.

**Method:**

Confirmatory factor analytic models were fitted to a clinical neurosciences archival data set (*n_*_Male_ = 166, *n_*_Female_ = 155) and to 9 age-group samples of the WAIS-IV standardization data (*n* = 200 for each group). The clinical and the standardization samples differed as (a) the former comprised scores from patients, aged 16 to 91, with disparate neurological diagnosis whereas the latter was demographically stratified, (b) only the 10 core subtests in the former but all 15 subtests in the latter were administered, and (c) the former had missing data, but the latter was complete.

**Result:**

Despite empirical constraints to eliciting 5 factors with only 10 indicators, the well-fitting, 5-factor (acquired knowledge, fluid intelligence, short-term memory, visual processing, and processing speed) measurement model evinced metric invariance between the clinical and standardization samples.

**Conclusion:**

The same cognitive constructs are measured on the same metrics in every sample examined and provide no reason to reject the assumption that the 5 underlying latent abilities of the 15 subtest version in the standardization samples can also be inferred from the 10 subtest version in clinical populations.

## Introduction

The Wechsler Adult Intelligence Scale–Fourth Edition (WAIS-IV) comprises 10 core and five supplemental subtests. The 10 core subtests are sufficient to compute the WAIS-IV cognitive indexes (Wechsler, 2008b). The supplemental subtests provide additional measures of cognitive functions and can be used as alternatives, if required, to calculate the indexes. Statistically robust factor analysis, providing psychometric validity evidence, typically requires a minimal ratio of 2–3 indicators or subtests per factor (Marsh, Hau, Balla, & Grayson, 1998). Based on the WAIS-IV standardization sample of the 15 subtest scores, a five-factor confirmatory factor analytic (CFA) model aligned with Cattell–Horn–Carroll (CHC) taxonomy of intelligence has been proposed (Benson, Hulac, & Kranzler, 2010; Lichtenberger & Kaufman, 2009; Sudarshan, Bowden, Saklofske, & Weiss, 2016). Scalar invariance of a five-factor model was established across the nine age groups, spanning 16–69 years (Sudarshan et al., 2016). Scalar invariance implies precise statistical generalization of the underlying latent variable model across all age groups.

The CHC model of cognitive ability is a hierarchical, three-stratum model. Stratum one comprises specific cognitive functions, termed the narrow abilities, typically associated with specific test responses. At stratum two, each broad ability equates with familiar cognitive constructs such as working memory and fluid reasoning, estimated from the respective test-specific narrow abilities. An overarching general intelligence “g,” which affects all the broad abilities to varying degrees, is at stratum three. Details of the CHC model are available in McGrew (2005) and Schneider and McGrew (2018).

The five CHC broad abilities associated with the WAIS-IV are (a) acquired-knowledge Gc, (b) fluid intelligence Gf, (c) visual processing ability Gv, (d) short-term memory capacity Gsm, and (e) processing speed Gs (Benson et al., 2010; Lichtenberger & Kaufman, 2009; Sudarshan et al., 2016).The WAIS-IV Indexes corresponding to Gc, Gsm, and Gs are Verbal Comprehension Index (VCI), Working Memory Index, and Processing Speed Index, respectively. The Perceptual Reasoning Index (PRI) of the WAIS-IV is a composite measure of reasoning (Gf) and visual processing ability (Gv). For a description of the subtests, indexes, and their relationships, refer to Wechsler (2008a).

**Fig. 1:**
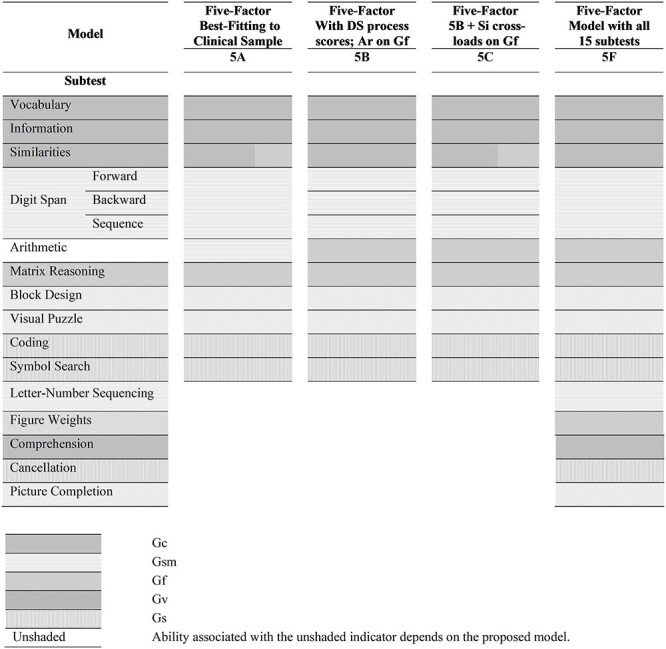
Association of indicators to latent constructs in the five-factor CFA measurement models underlying the WAIS-IV subtests. 5A is a model fitted to the scores in the core subtests. 5B and 5C are models fitted to the core subtests, but instead of composite Digit Span score, the three process scores are used. Model 5F is a model fitted to all the 15 subtests. Each row corresponds to an indicator and each column a model. The model cells are shaded as per the proposed factor(s) onto which the indicator loads. Cross-loading indicators in a model are split-shaded. The indicator cells are shaded as per the ability it predominantly reflects. Ar = Arithmetic; DS = Digit Span; Si = Similarities.

The core subtests and the full complement of subtests of the WAIS-IV are shown in Fig. 1. The subtests are listed in the first column, and each subtest is shaded in accordance with the predominant broad ability that is hypothesized to underlie responses to items in that subtest. This association of the subtests to the broad abilities is based on many past investigations (Benson et al., 2010; Sattler & Ryan, 2009; Sudarshan et al., 2016). Alternately, the first column can be viewed as a list of indicators that represent the subtest scores. Each of the other columns represents a model. The cells in the model columns represent factors onto which the indicator loads. The factors are labeled with the name of the ability or abilities they represent and are differentiated by their shading. The response to an item in Arithmetic involves quantitative reasoning, mathematical achievement, and working memory (Flanagan, McGrew, & Ortiz, 2000; Karzmark, 2009; Sattler & Ryan, 2009). Consequently different proposed models treat Arithmetic as an indicator of one or more of the factors that represent the broad abilities Gf, Gc, and Gsm (Benson et al., 2010; Bowden, Saklofske, & Weiss, 2011; Sudarshan et al., 2016; Ward, Bergman, & Hebert, 2012; Wechsler, 2008b; Weiss, Keith, Zhu, & Chen, 2013). All other subtests, as shown in Fig. 1, are associated with one CHC broad ability.

Although the scores from the 10 core WAIS-IV subtest span five broad abilities, a five-factor CFA model may not be empirically feasible when analyzed in the 10 subtest data set. The rule that recommends at least three indicators per factor, to avoid empirical under-identification and problems of non-convergence, limits models with 10 indicators to three factors (Kenny, 1979; Kline, 2011; Marsh et al., 1998). Prior studies, which fit CFA models to the 10 core subtest scores in the standardization data of the WAIS-IV, show that models with fewer than four factors fit poorly, but four-factor models fit well (Bowden et al., 2011; Wechsler, 2008b). In the models proposed by Bowden et al. (2011) and by Wechsler (2008b), the factor PRI represents a composite of the abilities Gv and Gf because the subtests associated with PRI span both these abilities (Flanagan et al., 2000; Sattler & Ryan, 2009). The underlying premise in such a model is that, with more suitable indicators, the composite factor will resolve into separate factors of respective abilities. Alternatively, a five-factor model may be estimated from the 10 subtest version if some subtests are allowed to cross-load to enable factor identification. Such a model has been fitted to the Wechsler Intelligence Scale for Children–IV (Thaler et al., 2015). However, cross-loadings created to achieve model identification complicate clinical and theoretical interpretation.

Another consideration in CFA modeling is the interpretability of the measures of the proposed latent factors. A factorially simple indicator is one that loads onto a single factor (McDonald, 1999). A correlated-factors model with an independent-clusters basis is one in which at least two indicators load solely on each factor, and such models facilitate theoretical interpretation (McDonald, 1999). Although the 10-indicator set spans all the five abilities, only one unidimensional indicator from this set loads on Gf or Gsm, namely, Matrix Reasoning and Digit Span, respectively. A model with a factor with a single indicator can be identified only by fixing the residual variance of that lone indicator to an arbitrary value (Bollen, 1989). Fixing the residual variance of an indicator arbitrarily prespecifies the reliability of the associated factor. Furthermore, it is unrealistic to expect equal residual variance across age groups (Widaman & Reise, 1997). So, it is not possible to establish a measurement model of the 10 core subtests that reflects the CHC taxonomy accurately. Therefore, estimating five-factor models in the 10-indicator data set would require arbitrary restrictions or cross-loadings. Within the 10 indicators, the only plausible indicator of Gsm, other than Digit Span, is Arithmetic. Prior research has established that cross-loading of Similarities on Gf or a Gf composite improves fit in some samples. An alternative estimation procedure follows Niileksela, Reynolds, and Kaufman (2013) who, in their analysis of standardization sample data on people older than 70, used the three separate parts of the Digit Span subtests as three different indicators for Gsm and associated Arithmetic solely with Gf.

Invariance testing can be used to investigate if the same underlying traits are being measured in two different populations (Meredith, 1993). Metric invariance, also referred to as weak invariance, establishes that the strength of association between the factor and the indicator that measures that factor, are the same across the groups. Metric invariance implies that the intervals in the scales of measurement of the underlying factors are the same across the groups. Scalar or strong invariance establishes that the zero points of the factors measured in the two groups coincide and the factor mean differences across groups are identified (Widaman & Reise, 1997). To evaluate invariance, the same factor model is fitted to samples from both the populations with increasing equality constraints, establishing (a) a configural invariance when the same model fits the samples adequately, (b) a metric invariance when further, the indicator loadings are fixed to be the same across the samples, and (c) a scalar invariance when, additionally, the intercepts are fixed to be the same across the samples (Meredith, 1993; Widaman & Reise, 1997).

The current study investigates whether a clinical sample in which only 10 subtests were routinely administered can be interpreted in the same manner as the five-factor model derived from the standardization sample. The clinical sample was obtained from patients aged from 16 to 91, with heterogeneous neurological diagnosis. The questions addressed in this study are: (a) What is the best-fitting model to the clinical sample? (b) Is there a generic factor model applicable to the standardization and the clinical samples, and by extension a common model for clinical and non-clinical population? (c) Can measurement invariance be established between the clinical and standardization samples to justify consistent test score interpretation across the clinical and non-clinical population? (d) Can any differences between clinical and non-clinical samples be derived from this modeling exercise?

## Method

### Participants

**Fig. 2:**
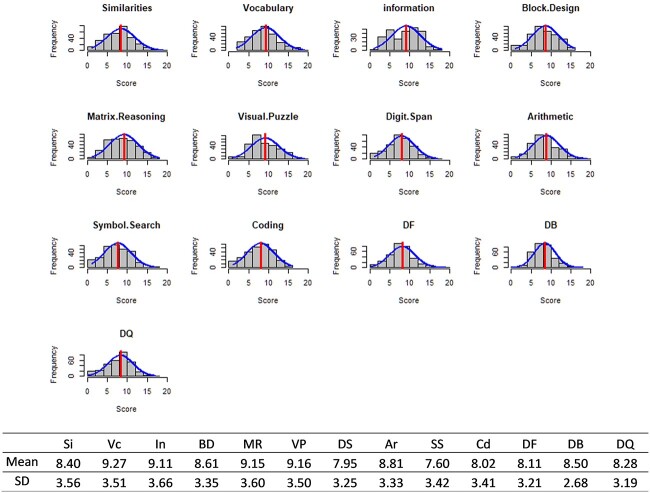
Distribution of subtest scores in the clinical sample*.* Ar = Arithmetic, BD = Block Design, Cd = Coding, DB = Digit Span Backward, DF = Digit Span Forward, DQ = Digit Span Sequencing, DS = Digit Span, In = Information, MR = Matrix Reasoning, Si = Similarities, SS = Symbol Search, Vc = Vocabulary, VP = Visual Puzzle. The histograms and the superposed normal curve were plotted using the routine plotNormalHistogram in Mangiafico (2016).

The clinical samples were obtained from non-identifiable archival data collected from patients with heterogeneous neurological conditions, referred to the Department of Clinical Neurosciences at St. Vincent’s Hospital, Melbourne for neuropsychological assessment. The study was approved by the St. Vincent’s Hospital Human Research and Ethics Committee. Each patient was tested by a registered psychologist or a research psychologist trained in the administration of the WAIS-IV. Three hundred and twenty-one cases were identified to have scores on the WAIS-IV subtests, of which 228 had no data missing. These participants were consecutive referrals to the neuropsychology service, for whom WAIS-IV data were available. Most patients were under the care of physicians in the Neurosciences Department, but some referrals came from general medical wards. This study was not preregistered. The distribution of scores in the clinical sample are shown in Fig. 2. The clinical data used in the study can be made available on request to the authors.

The standardization samples were selected to represent the US population, stratified across age, gender, ethnicity, educational level of self and parents, and region of residence. The demographics of the U.S. sample matched the 2005 U.S. census. The demographic characteristics and inclusion criteria of the samples can be found in the WAIS-IV Technical and Interpretive Manual (Wechsler, 2008b). The intercorrelations, means, and standard deviations of the subtest and process scores in the standardization data are available in appendix A of the WAIS-IV technical and interpretive manual, and were used in this analysis (Wechsler, 2008b).

### Instrument

The descriptions of the 15 subtests of the WAIS-IV are available in Lichtenberger and Kaufman (2009) and Wechsler (2008c). The clinical sample contained scores only from the 10 core subtests, and the analyses of the standardization samples were restricted to the scores from these core subtests. In two models, the process scores, Digit Span Forward, Digit Span Backward, and Digit Span Sequencing that contribute to the Digit Span score, were treated as indicators in their own right.

### Data analysis

Mplus version 7 was used to perform the CFA (Muthén & Muthén, 1998–2012). Maximum likelihood estimation was used with missing-at-random assumptions. The three models, described subsequently, were fitted to the clinical sample scaled scores and the nine age-group samples of scaled scores derived from the correlation matrices, means, and standard deviations reported in the manual. Further to the Mplus analysis, parameter estimates were checked for proximity to inadmissible values to anticipate any estimation problem in other samples. We also examined the estimated loadings for significance.

A plausible five-factor model with the core 10 indicators and which avoids fixing residual variances to arbitrary values is shown as Model 5A in Fig. 1. In this model, Arithmetic loaded on Gsm and Similarities cross-loaded on Gf and Gc. In many proposed five-factor models, Arithmetic loads on Gf (Benson et al., 2010; Lichtenberger & Kaufman, 2009; Sudarshan et al., 2016). In Models 5B and 5C, the Digit Span process scores—Digit Span Forward, Digit Span Backward, and Digit Span Sequencing—were treated as separate indicators in lieu of the composite digit span scores. In Model 5B, Arithmetic loaded on Gf, and Gsm was measured by each of the three process scores. In Model 5C, additionally, Similarities cross-loaded onto Gf.

Initially, all the models were fitted to the clinical sample and then to all the age-group standardization samples. The three five-factor models were tested for measurement invariance between the clinical sample and each of the nine age-group samples. To ensure that the fit of the five-factor model to the clinical sample was not fortuitous, we fitted the five-factor models to 500 repeated random subsamples of 200 cases, commensurate with the age-group sample sizes. This analysis was carried out using the MplusAutomation package in R to create the subsamples and control the repeated model fitting runs (Hallquist & Wiley, 2018).

In the test for invariance, the loss in fit by additional restrictions were gauged by the decrement in the fit measures comparative fit index (CFI) and McDonald’s non-centrality index (Mc). Whereas Cheung and Rensvold (2002) suggest decrements in excess of 0.01 in CFI and of 0.02 in Mc to reject the null hypothesis of invariance, Meade, Johnson, and Braddy (2008) recommend more stringent values of 0.002 for CFI, but offer no specific Mc cutoff recommendation for models with 10 indicators and more than 3 factors. The changes in fit indexes are compared against the configural baseline by Cheung and Rensvold, whereas Meade et al. compare the changes in fit at each step, namely, between configural and weak or between weak and strong invariance models, respectively.

## Results

Key results reported here are relevant for (a) the identification of the best-fitting model to the clinical sample, (b) the identification of a model that performs well across clinical and multiple age-group samples, (c) the invariance study across the clinical and non-clinical sample of the model most consistent with the best-fitting five-factor model on the full complement of the standardization sample subset scores, and (d) indicating possible difference between clinical and non-clinical samples in the abilities availed in responding to test items.

### Model fit

It can be seen in Fig. 2 that the mean scores in all the subtests for the clinical sample are less than 10, the normative scaled-score mean. The fit of the three five-factor models to the clinical samples are tabulated in Table 1. Model 5A wherein the Similarities cross-loaded onto Gf as well as Gc, and Arithmetic loaded on Gsm, was the best-fitting model. Warnings issued by the CFA software are recorded in the penultimate right column of Table 1 and the key parameter(s), if any, for which estimates are close to or violate statistical bounds, in the last column.

**Table 1:** Summary of fit measures for five-factor models fitted to the clinical sample

*χ* ^2^	*df*	CFI	TLI	RMSEA	SRMR	Sample size	Warning	Boundary value^a^
Model 5A. Similarities cross-loads on Gf, Arithmetic loads only on Gsm (best-fitting model on clinical data)								
48.592	24	0.987	0.975	0.056	0.023	321	None	None
Model 5B. Arithmetic loads only on Gf. Digit Span process scores load on Gsm								
103.845	44	0.972	0.958	0.065	0.033	321	None	None
Model 5C. Arithmetic loads only on Gf. Similarities cross-loads on Gf. Digit Span process scores load on Gsm								
89.381	43	0.978	0.967	0.058	0.031	321	Warning^b^	None

*Note*: The five-factor models are shown in Fig. 1. CFI = comparative fit index; *df* = degrees of freedom; RMSEA = root mean square of error of approximation; SRMR = standardized root mean square residual; TLI = Tucker–Lewis index. Warning refers to any issued by Mplus

^a^Entries in the Boundary value column refer to parameters whose estimated value was within 1.96 SE of a statistical boundary of admissible values or is the cause of inadmissible estimates of other parameters (refer to text for details).

^b^Mplus issued a warning when estimating the Model 5C, but no parameter was found to be within 2 SE of an inadmissible boundary value.

**Fig. 3:**
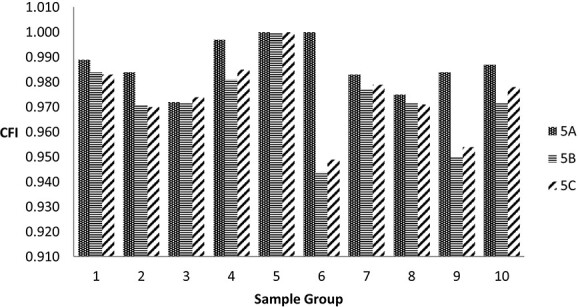
Comparison of CFI for Models 5A, 5B, and 5C fitted to the 9 age group and the clinical sample. The 9 age-group samples are numbered 1 to 9 (age span details in the text) and the clinical sample is numbered 10. In all three models, Vocabulary, Information, and Similarities loaded on Gc; Block Design and Visual Puzzles loaded on Gv; Coding and Symbol Search loaded on Gs. Matrix Reasoning loaded on Gf. In Model 5A, Digit Span and Arithmetic loaded on Gsm. In Models 5B and 5C, the three process scores Digit Span Forward, Digit Span Backward, and Digit Span Sequencing were treated as distinct indicators and loaded on Gsm, and Arithmetic loaded on Gf. In Models 5A and 5C, Similarities could cross-load onto Gf.

The measures of fit of Models 5A, 5B, and 5C to the various samples are shown in Table 2. The best-fitting model for the clinical sample, Model 5A, fitted well to all the age-group samples, but although the analysis of four age-group samples terminated normally, they were accompanied with warnings and the standard errors of some parameter estimates associated with Similarities loading on Gf were large. The cross-loading of Similarities improved the fit and estimation in the clinical sample and in some age-group samples, but not in the samples from age groups 16–17, 18–19, 30–34, and 55–64. As can be seen in Fig. 3, judging by fit alone, Model 5A is consistently superior.

**Table 2:** Summary of fit measures for five-factor models fitted to nine age-group samples in the U.S. standardization data for WAIS-IV and the clinical sample

Group	*χ* ^2^	*df*	CFI	TLI	RMSEA	SRMR	Sample size	Warning	Boundary value^a^
Model 5A. Similarities cross-loads on Gf, Arithmetic loads on Gsm (best-fitting model on clinical data)									
16–17 years	32.034	24	0.989	0.980	0.041	0.025	200	Warning	MR, Gf
18–19 years	39.735	24	0.984	0.970	0.057	0.028	200	None	Ar, Gf~Gv
20–24 years	52.96	24	0.972	0.947	0.078	0.032	200	None	Gf
25–29 years	27.511	24	0.997	0.994	0.027	0.024	200	None	None
30–34 years	23.612	24	1.000	1.001	0.000	0.021	200	Warning	Gf
35–44 years	24.418	24	1.000	0.999	0.009	0.021	200	None	Gf~Gv
45–54 years	42.237	24	0.983	0.967	0.062	0.027	200	None	Gf
55–64 years	50.276	24	0.975	0.954	0.074	0.031	200	Warning	Gf, MR
65–69 years	42.960	24	0.984	0.970	0.063	0.026	200	Warning	Gf
Clinical	48.592	24	0.987	0.975	0.056	0.023	321	None	None
Model 5B. Similarities cross-loads on Gf, Arithmetic loads on Gf. Digit Span process scores load on Gsm									
16–17 years	58.003	44	0.984	0.976	0.040	0.031	200	None	Gf~Gsm
18–19 years	76.468	44	0.971	0.956	0.061	0.045	200	Warning	None
20–24 years	76.183	44	0.972	0.958	0.060	0.036	200	Warning	Gf~Gsm, Gc
25–29 years	66.813	44	0.981	0.972	0.051	0.035	200	None	Gf~Gsm
30–34 years	41.845	44	1.000	1.003	0.000	0.026	200	Warning	Gf~Gsm
35–44 years	114.967	44	0.944	0.916	0.090	0.055	200	Warning	Gf~Gv
45–54 years	71.516	44	0.977	0.965	0.056	0.033	200	None	None
55–64 years	78.33	44	0.972	0.958	0.062	0.040	200	Warning	Gf~Gsm, Gv
65–69 years	113.659	44	0.950	0.924	0.089	0.050	200	None	None
Clinical	103.845	44	0.972	0.958	0.065	0.033	321	None	None
Model 5C. Similarities cross-loads on Gf, Arithmetic loads on Gf. Digit Span process scores load on Gsm									
16–17 years	57.776	43	0.983	0.974	0.041	0.031	200	None	Gf~Gsm, Si~Gf
18–19 years	76.423	43	0.970	0.953	0.062	0.045	200	Warning	Si~Gf, Gc
20–24 years	72.652	43	0.974	0.96	0.059	0.035	200	Warning	Gf~Gc
25–29 years	60.633	43	0.985	0.978	0.045	0.032	200	None	None
30–34 years	41.203	43	1.000	1.002	0.000	0.026	200	Warning	Gf~Gsm, Si~Gf, Gc
35–44 years	107.25	43	0.949	0.922	0.086	0.054	200	Warning	Gf~Gv
45–54 years	67.534	43	0.979	0.968	0.053	0.032	200	None	None
55–64 years	77.662	43	0.971	0.956	0.063	0.040	200	Warning	Gf~Gsm, Gv Si~Gf
65–69 years	106.762	43	0.954	0.929	0.086	0.048	200	None	None
Clinical	89.381	43	0.978	0.967	0.058	0.031	321	Warning	None

*Note*: Model structures are shown in Fig. 1. CFI = comparative fit index; *df* = degrees of freedom; MR = Matrix Reasoning; RMSEA = root mean square of error of approximation; SRMR = standardized root mean square residual; TLI = Tucker–Lewis index. Warning refers to any issued by Mplus

^a^Entries in the Boundary value column refer to parameters whose estimated value was within 1.96 SE of a statistical boundary of admissible values, or is the cause of inadmissible estimates of other parameters (refer to text for details): Ar = residual variance of Arithmetic; Gf = multiple parameters involving Gf; Gf~Gc = correlation between Gf and Gc; Gf~Gv = correlation between Gf and Gv; MR = residual variance of Matrix Reasoning; Si ~ = loading of Similarities on

### Parameter estimates

In addition to the fit comparisons, we examined the estimated parameters and the precision of the estimates in all the three five-factor models, to decide upon an acceptable common model to all the sample groups. Among the five-factor models, Model 5B, although not the best fitting, was the most acceptable of the models in the sense that in every group, none of the estimated parameter was inadmissible, although a few factor correlations were close to unity as seen in Table 3. We also examined the pairwise group differences in the parameter estimates for each model. The *t*-test results of comparing corresponding estimates at .05 significance level are tabulated in Table 4 for all the three five-factor models.

**Table 3:** Parameter value estimates for Model 5B fitted to nine age-group samples in the U.S. standardization data for WAIS-IV and the clinical sample

	Estimate	SE	Estimate	SE	Estimate	SE	Estimate	SE	Estimate	SE
	16–17 years		18–19 years		20–24 years		25–29 years		30–34 years	
Gc by										
VC	0.843	0.034	0.871	0.025	0.907	0.021	0.921	0.020	0.861	0.025
SI	0.700	0.044	0.810	0.031	0.822	0.028	0.849	0.026	0.807	0.030
IN	0.758	0.040	0.800	0.032	0.814	0.029	0.763	0.034	0.865	0.025
Gv by										
BD	0.825	0.038	0.790	0.038	0.808	0.040	0.847	0.037	0.806	0.036
VP	0.776	0.041	0.848	0.035	0.817	0.040	0.767	0.041	0.832	0.034
Gf by										
MR	0.629	0.051	0.652	0.048	0.614	0.051	0.648	0.049	0.735	0.039
AR	0.764	0.044	0.721	0.044	0.749	0.044	0.740	0.044	0.775	0.036
Gsm by										
DF	0.601	0.054	0.585	0.058	0.660	0.051	0.707	0.044	0.683	0.046
DB	0.714	0.046	0.738	0.048	0.683	0.050	0.749	0.040	0.772	0.039
DQ	0.716	0.045	0.701	0.050	0.714	0.048	0.766	0.039	0.705	0.044
Gs by										
SS	0.611	0.073	0.801	0.046	0.801	0.051	0.838	0.045	0.757	0.046
CD	0.753	0.077	0.824	0.045	0.799	0.051	0.764	0.046	0.845	0.044
GV with										
Gc	0.675	0.058	0.714	0.050	0.633	0.057	0.625	0.057	0.666	0.054
Gf with										
Gc	0.724	0.064	0.861	0.053	0.930^a^	0.048	0.851	0.051	0.848	0.044
Gv	0.834	0.060	0.872	0.058	0.836	0.061	0.861	0.058	0.913	0.044
Gsm with										
Gc	0.607	0.067	0.485	0.073	0.596	0.064	0.655	0.055	0.690	0.054
Gv	0.678	0.065	0.380	0.083	0.631	0.067	0.663	0.061	0.678	0.060
Gf	0.956^a^	0.056	0.868	0.064	0.886^a^	0.062	0.895^a^	0.055	0.912^a^	0.046
Gs with										
Gc	0.289	0.091	0.567	0.063	0.488	0.068	0.486	0.068	0.524	0.066
Gv	0.496	0.086	0.535	0.069	0.522	0.072	0.607	0.066	0.539	0.070
Gf	0.593	0.090	0.746	0.067	0.668	0.074	0.660	0.073	0.722	0.062
Gsm	0.528	0.088	0.453	0.080	0.555	0.074	0.672	0.061	0.698	0.060
	**Estimate**	**SE**	**Estimate**	**SE**	**Estimate**	**SE**	**Estimate**	**SE**	**Estimate**	**SE**
	**35–44 years**		**45–54 years**		**55–64 years**		**65–69 years**		**Clinical**	
Gc by										
VC	0.899	0.022	0.892	0.022	0.922	0.019	0.890	0.021	0.875	0.019
SI	0.834	0.028	0.823	0.028	0.823	0.027	0.831	0.027	0.882	0.019
IN	0.788	0.032	0.815	0.029	0.830	0.027	0.798	0.030	0.812	0.025
Gv by										
BD	0.837	0.035	0.846	0.038	0.823	0.040	0.840	0.033	0.874	0.023
VP	0.824	0.035	0.792	0.040	0.741	0.043	0.786	0.036	0.855	0.025
Gf by										
MR	0.710	0.042	0.717	0.041	0.702	0.044	0.729	0.038	0.754	0.030
AR	0.719	0.042	0.795	0.036	0.684	0.045	0.837	0.030	0.779	0.031
Gsm by										
DF	0.669	0.048	0.695	0.046	0.515	0.059	0.699	0.045	0.694	0.036
DB	0.790	0.039	0.808	0.039	0.702	0.045	0.860	0.034	0.795	0.029
DQ	0.718	0.044	0.657	0.049	0.819	0.037	0.647	0.049	0.804	0.028
Gs by										
SS	0.790	0.047	0.731	0.052	0.787	0.043	0.801	0.041	0.847	0.025
CD	0.836	0.046	0.875	0.051	0.838	0.041	0.799	0.041	0.893	0.023
GV with										
Gc	0.632	0.056	0.618	0.058	0.593	0.061	0.769	0.045	0.662	0.042
Gf with										
Gc	0.879	0.047	0.845	0.043	0.868	0.050	0.919	0.033	0.893	0.032
Gv	0.907^a^	0.050	0.802	0.053	0.915^a^	0.058	0.892	0.042	0.896	0.034
Gsm with										
Gc	0.633	0.058	0.628	0.058	0.693	0.053	0.675	0.053	0.701	0.040
Gv	0.537	0.069	0.543	0.070	0.585	0.070	0.577	0.066	0.657	0.046
Gf	0.881	0.054	0.837	0.052	0.961^a^	0.054	0.766	0.052	0.854	0.039
Gs with										
Gc	0.431	0.071	0.526	0.066	0.517	0.065	0.611	0.060	0.708	0.039
Gv	0.467	0.073	0.522	0.070	0.665	0.062	0.692	0.059	0.769	0.038
Gf	0.722	0.066	0.634	0.068	0.768	0.065	0.755	0.055	0.823	0.039
Gsm	0.582	0.067	0.506	0.073	0.622	0.065	0.664	0.060	0.756	0.040

*Note*: Model 5B is shown in Fig. 1. The age-group sample sizes = 200 and that of clinical data = 321

^a^Parameter estimate is not significantly different from one.

### Invariance testing

The results of testing for measurement invariance of Model 5B between the clinical sample and each of the age-group samples are shown in Table 5. In the invariance estimations, no parameter had an improper value, but consistent with the single group estimations shown in Table 3, the factor correlations of Gf, especially with Gsm, were within two SEs of unity for several age groups. We note that metric invariance (equality of factor loadings) held in every comparison, but strong invariance (additional equality of subtest intercepts) failed in almost every comparison when judged by criteria suggested by Cheung and Rensvold (2002). In the age groups above 45 years, the weak invariance was rejected by the criteria stipulated by Meade et al. (2008) because the incremental CFI exceeded 0.002. This finding indicates that the intercepts of the regression relationship between the indicators and the factors differ, but the slopes are the same in the groups compared.

### Fit in clinical subsamples

The results of repeated clinical subsample runs for the five-factor models are shown in Table 6. In the estimation of Model 5A, 20% of the runs had sample-specific parameter estimation or admissibility problems. No problems were encountered during the estimation of fit and parameters for Models 5B and 5C. The average CFI and root mean square of error of approximation (RMSEA) match the CFI and RMSEA obtained when the model was fitted to the entire clinical sample (compare Table 1 and Table 6). However, the respective chi-square values are different, reflecting the sensitivity of chi-square estimations to sample size (Bentler & Bonett, 1980).

**Table 4:** Between-group comparisons of estimates of factor loading when the five-factor model fitted to the clinical and the age-group samples

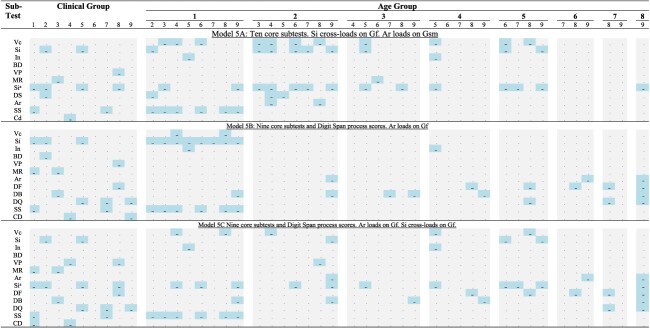

*Note*. Five-factor models are shown in Fig. 1. Groups in top header row were compared with each of the corresponding groups in the bottom header row. The difference between estimates of corresponding loadings were tested for significance level of .05. The shaded and unshaded entries indicate significant and non-significant differences, respectively. Ar = Arithmetic, BD = Block Design, Cd = Coding, DB = Digit Span Backward, DF = Digit Span Forward, DQ = Digit Span Sequencing, DS = Digit Span, In = Information, MR = Matrix Reasoning, Si = Similarities, SS = Symbol Search, Vc = Vocabulary, VP = Visual Puzzle

^a^Refers to the secondary loading of Similarities on Gf

### Similarities cross-load estimates

We note, in Table 7, that some age groups consistently show significant cross-loadings of Similarities on Gf across the models. The cross-loading of Similarities affected the estimations of parameters associated with Gf and the loading of Similarities on Gc (refer Table 2, Warning and Boundary value columns).

In summary, the results show that (a) the five-factor models fitted the clinical sample very well and were estimated without any issues; (b) there was substantial improvement of model fit to the clinical sample when Similarities was allowed to cross-load on Gf; (c) in contrast to significant cross-loading of Similarities seen in models fitted to the clinical sample, the estimated cross-loading of Similarities for some of the standardization age-group samples were not significant; (d) for these models, the estimates of some parameters related to Gf in some age-group samples were inadmissible or close to inadmissibility, (e) but even in these instances, the analysis terminated normally and the fit measures were good; (f) that in most pairwise group comparisons, the corresponding estimated factor loading did not differ significantly; and (g) metric invariance can be established between the clinical sample and each of the age groups, but scalar invariance tests failed.

## Discussion

In isolation, Model 5A was the best-fitting model in the clinical sample. Multiple models were fitted to several non-clinical samples, namely, the age-cohort standardization samples, and to the clinical sample to investigate the possibility of a common model, so that score interpretation is consistent among the groups. The non-clinical samples were expected to provide a coherent contrast to the clinical sample because scalar invariance had been established among these standardization samples (Sudarshan et al., 2016). For each model, we scrutinized the fit and the relationship between the subtest scores and the ability constructs for any differences between the clinical sample and the standardization samples. The principal difference we found was that in the clinical sample, the cross-loading of Similarities to Gf significantly improved the fit although in many of the standardization samples, the improvement in fit associated with the imposition of this cross-loading was marginal, and the precision of parameter estimates related to Gf deteriorated, leading to inadmissible parameter estimates. However, the cross-load did improve, albeit with a drop in the precision of the estimate, the model fit to the samples from age groups 3, 4, 6, 7, and 9. Mulaik (2009) points out that an inherently low loading by itself is not a reason to reject a model. Responses, both in Matrix Reasoning and in Similarities, require the induction of organizing principles in the stimuli (Kaufman & Lichtenberger, 2005). This common requirement may plausibly explain the improvement in fit, where it occurs. However, in the context of multiple samples where the cross-loading results in secondary estimation issues, it may be prudent to ignore this cross-loading. The cross-loading of Similarities can be considered as a characteristic of the test, but is relatively unimportant for interpreting test scores within the CHC framework. As a consequence, Model 5B was preferred as better fitting in all the sample, in preference to Model 5A.

A CFA study limited to core subtests cannot support, in entirety, the validity of interpreting the WAIS-IV test scores as measures of the underlying cognitive abilities. We know that the statistical estimation results with the complete 15 indicator set are comparable among the age-group samples, and so any differences in the outcomes with the diminished indicator set between the various age-group samples may be attributed to the approximation forced by the diminished indicator set (Sudarshan et al., 2016). If the parameter estimates from the clinical sample lie within the range of parameter estimates in the standardization samples, then it can be surmised that there is no evidence that the clinical sample is from a different population of cognitive ability constructs.

**Table 5:** Goodness-of-fit statistics for evaluation of measurement invariance (metric and scalar) of Model 5B between nine of the age groups in the Wechsler Adult Intelligence Scale–IV: U.S. standardization sample and the clinical sample

	*χ* ^2^	*df*	CFI	ΔCFI	TLI	RMSEA			SRMR	Mc	ΔMc
						Value		90% CI			
16–17 years vs. clinical sample											
Configural invariance	161.849	88	0.976		0.963	0.057		0.043–0.070	0.024	0.932	
Metric invariance	169.505	95	0.975	-0.001	0.966	0.055		0.041–0.068	0.031	0.931	-0.001
Scalar invariance	205.056	102	0.966	-0.009	0.956	0.062		0.050–0.074	0.041	0.906	-0.025
18–19 years vs. clinical sample											
Configural invariance	180.307	88	0.972		0.957	0.063		0.050–0.076	0.025	0.916	
Metric invariance	186.733	95	0.972	0.000	0.961	0.061		0.048–0.074	0.035	0.916	0.001
Scalar invariance	211.603	102	0.966	-0.006	0.956	0.064		0.052–0.076	0.048	0.901	-0.016
20–24 years vs. clinical sample											
Configural invariance	180.027	88	0.972		0.958	0.063		0.050–0.076	0.027	0.916	
Metric invariance	187.377	95	0.972	0.000	0.961	0.061		0.048–0.074	0.031	0.915	0.000
Scalar invariance	209.867	102	0.967	-0.005	0.958	0.064		0.051–0.076	0.038	0.902	-0.013
25–29 years vs. clinical sample											
Configural invariance	170.664	88	0.975		0.963	0.060		0.046–0.073	0.023	0.924	
Metric invariance	185.005	95	0.973	-0.002	0.963	0.060		0.047–0.073	0.036	0.918	-0.006
Scalar invariance	215.402	102	0.966	-0.007	0.956	0.065		0.053–0.077	0.044	0.897	-0.020
30–34 years vs. clinical sample											
Configural invariance	145.687	88	0.983		0.975	0.050		0.035–0.064	0.022	0.946	
Metric invariance	153.048	95	0.983	0.000	0.976	0.048		0.034–0.062	0.033	0.946	0.000
Scalar invariance	177.41	102	0.978	-0.005	0.971	0.053		0.040–0.066	0.040	0.930	-0.016
35–44 years vs. clinical sample											
Configural invariance	218.816	88	0.962		0.943	0.075		0.063–0.088	0.022	0.882	
Metric invariance	228.001	95	0.961	-0.001	0.946	0.073		0.061–0.085	0.029	0.881	-0.002
Scalar invariance	256.74	102	0.955	-0.006	0.941	0.076		0.065–0.088	0.037	0.862	-0.018
45–54 years vs. clinical sample											
Configural invariance	175.358	88	0.974		0.961	0.062		0.048–0.075	0.024	0.920	
Metric invariance	198.017	95	0.969	-0.005	0.957	0.064		0.052–0.077	0.040	0.906	-0.014
Scalar invariance	224.826	102	0.963	-0.006	0.953	0.068		0.056–0.080	0.051	0.889	-0.017
55–64 years vs. clinical sample											
Configural invariance	182.175	88	0.972		0.958	0.064		0.051–0.077	0.026	0.914	
Metric invariance	198.052	95	0.969	-0.003	0.958	0.064		0.052–0.077	0.040	0.906	-0.008
Scalar invariance	230.107	102	0.962	-0.007	0.951	0.069		0.057–0.081	0.051	0.885	-0.021
64–69 years vs. clinical sample											
Configural invariance	217.501	88	0.963		0.945	0.075		0.063–0.088	0.024	0.884	
Metric invariance	236.406	95	0.960	-0.003	0.944	0.075		0.063–0.088	0.032	0.874	-0.010
Scalar invariance	253.487	102	0.957	-0.003	0.945	0.075		0.064–0.087	0.046	0.865	-0.008

*Note*: Sample size in each age group = 200, and in the clinical sample = 321*.* CFI = comparative fit index; *df* = degrees of freedom; Mc = McDonald’s non-centrality index; RMSEA = root mean square of error of approximation; SRMR = standardized root mean square residual; TLI = Tucker–Lewis index; ΔCFI = incremental change in CFI; ΔMc = incremental change in McDonald’s non-centrality index

**Table 6:** Summary statistics of fit measures of five-factor models fitted to random subsamples of the scores from the clinical data

Model	*df*	No. of runs		Mean	SD	Min	Max
Model 5A: 10 core subtest indicators			*χ* ^2^	40.018	7.141	21.695	62.400
Ar loads on Gsm	24	446	RMSEA	0.056	0.014	0	0.090
Si cross-loads on Gf (All completed runs)			CFI	0.986	0.006	0.968	1
Model 5A: 10 core subtest indicators			*χ* ^2^	39.943	7.194	21.695	62.400
Ar loads on Gsm	24	406	RMSEA	0.056	0.014	0	0.090
Si cross-loads on Gf (Runs without warnings)			CFI	0.986	0.006	0.968	1
Model 5B: indicators include			*χ* ^2^	83.128	11.711	51.851	124.500
Digit Span process scores	44	500	RMSEA	0.066	0.01	0.03	0.096
All runs terminated without warning			CFI	0.971	0.009	0.942	0.994
Model 5C: indicators include			*χ* ^2^	73.569	10.981	43.817	102.748
Digit Span process scores	43	500	RMSEA	0.059	0.011	0.010	0.083
Si cross-loads on Gf All runs terminated without warnings			CFI	0.977	0.008	0.954	0.999

*Note*. No. of subsamples = 500. Subsample size = 200. Ar = Arithmetic; Si = Similarities

**Table 7:** Secondary loading of Similarities in five-factor models in different sample groups

Model	5A: 5 factors, Ari loads on Gsm, Si cross-loads on Gf			5C: 5 factors, DS process scores load on Gsm, Ari loads on Gf, Si cross-loads on Gf		
Sample group	Estimate	SE	CFI	Estimate	SE	CFI
16–17 years	0.009^*^	0.136	0.989	−0.055^*^	0.111	0.983
18–19 years	−0.346^*^	0.197	0.984	−0.026^*^	0.106	0.970
20–24 years	0.387	0.128	0.972	0.243	0.116	0.974
25–29 years	0.216	0.081	0.997	0.247	0.094	0.985
30–34 years	−0.112^*^	0.119	1.000	−0.094^*^	0.119	1.000
35–44 years	0.265	0.072	1.000	0.255	0.086	0.949
45–54 years	0.192	0.097	0.983	0.223	0.102	0.979
55–64 years	0.068^*^	0.083	0.975	0.076^*^	0.089	0.971
65–69 years	0.368	0.120	0.984	0.403	0.134	0.954
Clinical	0.295	0.067	0.987	0.332	0.077	0.978

*Note*. Models 5A and 5C are shown in Fig. 1. The fit details of all the five-factor models are available in Table 2. Ari = Arithmetic; DS = Digit Span; Si = Similarities

^^*^^ *p* < .05

Two classes of approximation are forced when the number of indicators are limited. In the four-factor models of WAIS-IV scores, reported in prior clinical studies of the 10 subtest data sets, the statistical discrimination between the five factors is diminished (Bowden et al., 2011; Wechsler, 2008b). Estimation of the five-factor models in a clinical 10-subtest data set preserves the distinction between the five broad abilities, but requires freeing up parameters of secondary interest (like cross-loadings) or using constituent indicators in lieu of composite scores. The fit of a more complex model is likely to be better but more susceptible to “improper solutions” during estimation (Brown, 2006; McDonald, 1989). The empirically observed consequence of decreased degrees of freedom is an increase in estimation failures.

Therefore, overall Model 5B was judged the best-fitting model, when all the estimation outcomes are considered when fitted to all of the samples. The fit of Model 5B were reasonably good, CFI ranging from 0.942 to 1. No parameter estimate was inadmissible, albeit the factor correlations between Gf and Gsm in some samples were close to unity, which was not inconsistent with other findings that Gsm and Gf are highly correlated (Blair, 2006). In addition, the precision of estimates of the parameters were reasonable, the SEs ranging from 0.019 to 0.091 (refer to Table 3). More importantly, Model 5B is in alignment with the five-factor model for which measurement invariance was established between all the age-group samples in the standardization data, where the full complement of 15 subtests were administered (Benson et al., 2010; Lichtenberger & Kaufman, 2009; Sudarshan et al., 2016).

### Comparison of parameter estimates across groups

Model selection studies based on a single sample concentrate on fit and generally ignore the examination of estimated parameter values. In this multi-sample comparison, we explored if parameter values can be used to differentiate between samples, specifically if the loading patterns found in the clinical sample differed from those in the standardization sample.

The *t*-test for null difference between corresponding loadings at 0.05 significance level between every pair of sample groups is a heuristic approach to assess if the loadings in the clinical sample fell within the ranges observed in the age-group samples. As can be seen in Table 4, even within the age-group samples, there are instances of rejected null hypothesis. Visual inspection suggests that the parameter estimates match between clinical samples and age-group samples were worse than that among the age-group samples.

### Invariance study

Invariance analysis is a more rigorous approach to determine equality of numerical details of the factor model in every group. The invariance analysis found that Model 5B was metrically (factor-loading) invariant across the clinical group and the age cohorts, but scalar equivalence was not established. This finding indicates that the score differences within each group can be interpreted in like manner, but the scores differ across the clinical and the non-clinical samples. This finding is not surprising because the scaled scores are standardized to have a mean of 10 in each of the age cohorts. In contrast, the scaled scores in the clinical sample were the observed scores among patients, many of whom had neurological or medical diagnoses, and associated lower scores. Note in Fig. 2 that although scores in all the subtests in the clinical sample are lower, scores on Coding and Symbol Search, which are indicators of Gs, and those on Digit Span Forward and Digit Span Sequencing associated with Gsm are particularly low.

The emphasis in our invariance study was not to explore differences between the clinical and standardization groups, such as group means in a latent ability, but to assess the extent to which the parameters in the two groups are comparable. Imposition of weak invariance constraint is a statistically sound way to establish loading equivalence between the two groups. The clinical implication of this finding is that convergent and discriminant validity research can be conducted in an uncomplicated way when the five-factor scoring model is applied in clinical samples, and the results compared with community control samples.

The improvement in fit with the cross-loading of Similarities in clinical group warrants a more detailed study. Studies of serial assessment for cognitive decline in clinical population indicate that VCI scores are the most stable and Similarities subtest are the least stable over repeated assessments (Holdnack, Drozdick, Iverson, & Chelune, 2013). Weiss, Saklofske, Coalson, and Raiford (2010) suggest that a patient’s response to items in Similarities involve working memory, retrieval from long-term storage, and fluid reasoning. One possibility is that patients in the clinical group when responding to Similarities items are less able than community controls to use over-learned categorized information and compensate with ad hoc reasoning strategies. A more targeted study is required for assessing the relative impact of different cognitive abilities on the Similarities score.

In summary, the previous published studies fitting five factors to 15 indicators (Benson et al., 2010; Lichtenberger & Kaufman, 2009; Sudarshan et al., 2016) help one to look beyond the structural compromises imposed by the reduction in the number of indicators and concentrate on plausible structural and relational changes inherent to a different population. Our results show that the interpretation of the 10 subtest scores in terms of the five broad abilities based on the standardization sample models cannot be rejected as an implausible interpretation of the 10 subtest version in a clinical sample and should be used to guide clinical interpretation.

## Conflict of interest

There are no conflict of interest in this work for both the authors. Stephen Bowden receives royalty from Oxford University Press, and Editorial Stipend from Springer-Nature.

## Author contributions

Jason Sudarshan (Conceptualization, Data curation, Formal analysis, Investigation, Methodology, Writing—original draft), Stephen Bowden (Conceptualization, Methodology, Project administration, Supervision, Writing—review & editing).
